# Health allowance for improving the nutritional status and development of 3–5-year-old left-behind children in poor rural areas of China: study protocol for a cluster randomised trial

**DOI:** 10.1186/s13063-015-0897-5

**Published:** 2015-08-18

**Authors:** Qian Lin, Peymané Adab, Karla Hemming, Lina Yang, Hong Qin, Mingzhi Li, Jing Deng, Jingcheng Shi, Jihua Chen

**Affiliations:** School of Public Health, Central South University, 110 Xiangya Road, Changsha, 410078 Hunan China; Department of Public Health, Epidemiology & Biostatistics, University of Birmingham, Birmingham, UK

**Keywords:** CCT, Development, Left-behind children, Nutrition education, Undernutrition

## Abstract

**Background:**

Left-behind children (LBC) are recognised as a new social group in China. LBC are young children who are abandoned in rural villages whilst their parents travel to distant urban centres for employment (a new generation of migrant workers). Following the rapid growth in the number of migrant workers, the LBC population is also rapidly increasing. These children are usually left to be raised by elderly grandparents, a single parent, or sometimes distant relatives or neighbours who have limited resources, tend to have a poor education and sometimes are in frail health. Over 40 % of the 61 million LBC in China who are under 5 years old are undernourished, which affects their long-term health and abilities. An intervention that combines a conditional cash transfer (CCT) with nutrition education offers a potential solution.

**Methods/Design:**

A cluster randomised controlled trial design will be used to allocate 40 villages to the intervention arm (20 villages) or control arm (20 villages). The caregivers and all of the 3–5-year-old LBC will be the target population. Caregivers in the intervention arm will receive a cash allowance conditional on attending nutrition education sessions, ensuring that the LBC will use basic public health services over a 12-month period. At the baseline, midterm (month 6) and end (month 12) of the intervention period, evaluations will be conducted in all 40 villages. Multilevel generalised linear models will be used to analyse the impact of the intervention on nutrition status and other outcomes, adjusting for baseline levels using an analysis of covariance approach. The cost of the intervention will also be estimated.

**Discussion:**

If found to be cost-effective, the findings will inform the development of a sustainable model to improve nutrition status among LBC in rural areas of China.

**Trial registration:**

Chinese Trial Register (ChiCTR) identifier: CTXY-140003-2. Registered on 19 Aug 2014.

## Background

Left-behind children (LBC) are recognised as a new social group in China. They are young children in rural villages whose parents travel to distant urban centres to find employment (a new generation of migrant workers) [1]. Most LBC reside in the more populous but less developed regions of central and western China, including the rural areas of Sichuan, Hunan, Henan, Anhui, and Guangdong provinces. Following the rapid growth in the number of migrant workers, the LBC population is also sharply increasing. It is estimated that 61 million children in China fall into this category (one-fifth of all children in the country) and that over 40 % are younger than 5 years old and separated from both parents. This young subpopulation is also growing; it increased from 15.8 million in 2005 to 23.4 million in 2013 [2]. A national report indicated that undernutrition is particularly prominent in this particular group, which affects their long-term health and abilities.

Separated from parents, especially their mothers, LBC’s dietary intake and nutrition status, as well as attendance to their health needs, are poorer than for other children. LBC are usually left to be raised by elderly grandparents, a single parent, or sometimes distant relatives or neighbours who have limited resources, tend to have a poor education and sometimes are in frail health. Approximately one-half of the LBC are separated from both parents (i.e., both parents migrated), and one-third are cared for by elderly grandparents [1]. LBC frequently are undernourished owing to the poor living conditions and low education levels of their caregivers. A study of 11,231 rural children showed that parental migration from rural to urban areas had a significant negative effect on the nutrition and health of young LBC. Their growth Z-scores and consumption of dietary energy and nutrients were lower than those of other children [3]. Another survey, done in Hunan, showed insufficient energy intake in more than 50 % of LBC; insufficient protein intake in over 80 %; and insufficient intake of calcium, zinc, riboflavin and thiamine in over 90 % [4]. Two common manifestations of undernutrition in children younger than 5 years old are stunting and iron deficiency anaemia (IDA).

The Chinese government has implemented several policies and programmes to improve the nutrition status of LBC, including the Integrated Nutrition Package for rural infants (targeting children younger than 3 years old), Nutrition Education for Infant Feeding Practice and giving priority to LBC in the Nutrition Improvement Programme for school-age children (aged 6 years or older) [5–10]. The group of children aged 3–6 years old were previously neglected in the National Public Health Service Standard. Furthermore, there are no nutrition policies and few nutrition programmes targeting 3–5-year-old LBC. Currently, the undernutrition of LBC younger than 5 years old is a public health focus in China. The reduction of their stunting and IDA are also targets of the Chinese Children’s Development Programme (2011–2020).

Although the government provides health services for LBC in rural areas, access to and use of these services (including health checkups, growth monitoring and vaccination) has received little attention. Anecdotal evidence suggests that LBC are rarely brought in for appointments.

Conditional cash transfer (CCT) programmes are increasingly used as policy strategies to improve health, nutrition and education. These programmes, with financial incentives conditional on the receivers’ actions, have been planned or implemented in 33 countries. The results of systematic reviews suggest that the CCT programmes could increase food expenditure, improve education and increase health service use and thus reduce inequities in nutrition and health among poor children [11–16]. Mexico’s Oportunidades programme was a successful CCT programme. It provided evidence of increased height for age and decreased stunting, increased intake of key micronutrients and improved language development and cognitive performance in children whose families received the CCT intervention [17–20]. However, not all of the evaluations were based on a comparison of groups allocated randomly to a control or intervention arm. Furthermore, the effects of such an intervention in China may differ, as rural caregivers of LBC there may have less health and nutrition knowledge than elsewhere [21]. Studies done in other developing countries also suggest that a comprehensive nutrition intervention programme for rural children should include interventions to improve the household economy and knowledge of child nutrition and personal hygiene [22–24].

Currently, the undernutrition of LBC younger than 5 years old is a public health focus in China. The reduction of their stunting and IDA are also targets of the Chinese Children’s Development Programme (2011–2020). Although formal economic analyses have not been performed to date, there is some indication that such programmes are cost-effective in the long term and would therefore be fiscally sustainable interventions [25]. Evidence drawn from previous programmes suggests that, although these programmes entail great initial costs, the overall costs are marginal compared with those of other public health programmes. For example, the government embarked on an effort to improve children’s nutrition status by allocating 3 Chinese yuan renminbi (RMB) per child per meal (9 RMB/day) for 23 million primary and middle school students (6–15 years of age) in poor rural areas in 2011, which equates to 1530 RMB per child per year. The gap in nutrition between 3–5-year-old LBC and other children is widening.

## Aims and objectives

The aim of this study is to assess the clinical efficacy and cost-effectiveness of a CCT intervention, together with nutrition education, among caregivers of 3–5-year-old LBC in rural China and to assess the development and nutrition status of the LBC. The specific objectives include the following:

1. Comparison of the effectiveness of the intervention with no intervention (care as usual) regarding 3–5-year-old LBC’s nutrition status, including the prevalence of stunting and IDA
2. Assessment of the effects of the intervention on basic public health service use by LBC, including attendance at health checkups and vaccination and growth monitoring appointments
3. Assessment of the effect of the intervention on food expenditure and dietary diversity among 3–5-year-old LBC
4. Comparison of the cost-effectiveness of the intervention with no intervention (care as usual)

## Methods/Design

### Design and theoretical model

**Fig. 1:**
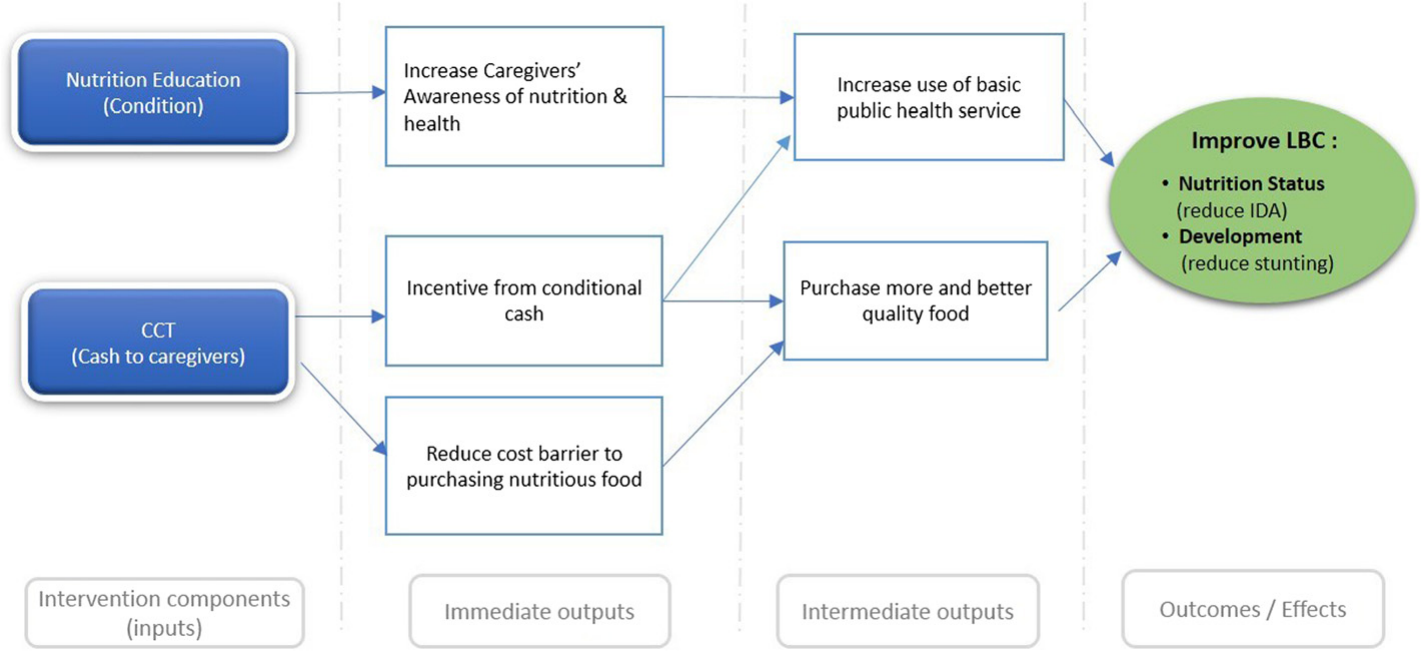
Logic model illustrating the theoretical pathway leading from intervention to improved health outcomes for 3–5-year-old left-behind children. *IDA* iron deficiency anaemia, *LBC* left-behind children

This study will be a cluster randomised controlled trial aimed at reducing the prevalence of stunting and IDA among young LBC in rural China. The theoretical basis for the intervention being tested is summarised in Fig. 1. We hypothesised that the intervention will affect the nutrition status and development of LBC through the following two pathways:

1. *CCT pathway*: Cost of food is a significant barrier to nutrition. The CCT will have an immediate effect on household food security, increasing the ability of caregivers to purchase more and better food (improving diet quality and diet quantity).
2. *Nutrition education pathway*: Low levels of education and poor health knowledge of the caregivers are additional barriers to health and nutrition among LBC. The CCT will give caregivers an incentive to attend the nutrition workshops (the “condition”). The aim of the workshops is to raise awareness among caregivers, who are the main decision-makers for a LBC’s health care and dietary arrangement. The workshops will cover the importance of early child nutrition and use of basic public health services (e.g., immunizations and health checks). By increasing the awareness and understanding of caregivers, we hypothesise that the quality of food offered to LBC will improve and that use of basic public health services will increase.

### Setting and participants

In Hunan Province, located in south-central China, more than one-half of the resident children are LBC. Xiangxi Tujia and Miao Autonomous Prefecture and Yiyang City will be chosen as the settings for this research project. Xiangxi Tujia and Miao Autonomous Prefecture is an impoverished mountainous region in western Hunan Province. These distinct locations are chosen to represent a range of geographic diversity for this project. Within the cities, Anhua County in Yiyang and Fenghuang County in Xiangxi, both nationally designated as poor and with a high LBC population (20,000–35,000) are chosen as the sites for this study. All 340 villages in Fenghuang County and 432 villages in Anhua County will form the overall sampling framework (clusters) and will be assessed for eligibility.

### Eligibility criteria for participants

We will recruit 20 eligible villages in Fenghuang County and 20 in Anhua County. The villages will be randomly selected if they meet the criteria described below:

1. *Inclusion criteria*: The villages will be included if they have a minimum of 15 LBC (3–5 years old) living in a poor household (defined as annual income less than RMB 2300) and have no kindergarten or care centre for LBC.
2. *Exclusion criteria*: Villages receiving other similar funding or benefits from other sources, such as charities or non-governmental organisations (NGOs) will be excluded.

Households in the intervention arm villages must meet the following eligibility criteria to qualify for cash support:

1. Caring for at least one left-behind child (3–5 years old)
2. ‘Poor household’, defined as per-capita annual income lower than RMB 2300
3. Not receiving benefits from a charity, NGO or other, similar programme

### Recruitment

With assistance from local health professionals, the research team will identify eligible subjects. The caregivers of eligible LBC will be approached and informed of the study. The procedure of the study and the implications of routine blood examination and anthropometric measurements will be explained to all of the caregivers. Informed consent will be sought by explaining to potential participants, in a language that the person can understand, the goal of the study, the procedure or activity and the risks and benefits of participation. These individuals will also be given the opportunity to ask questions. If inclusion criteria are met, written informed consent will be obtained from caregivers willing to enrol the LBC in the study.

### Ethical approval

This study is approved by the independent ethics committee of the Institute of Clinical Pharmacology, Central South University (project number CTXY-140003-2, July 2014). Written informed consent will be obtained from the caregivers. All participant data gathered in this study will be kept strictly confidential.

### Intervention

#### Development of nutrition education materials

The workshop will involve group learning for caregivers. The aim of the sessions will be to provide health and nutrition information, focusing on children’s needs, and to provide instructions on hygiene (e.g., hand-washing). Each workshop will have a main focus, and the topics covered will be as follows:

- Workshop 1: Introduction to the Basic Public Health Service for Left-Behind Children
- Workshop 2: Importance of Health Examinations for Left-Behind Children
- Workshop 3: Importance of Nutrition and Diet Diversity for Left-Behind Children’s Health
- Workshop 4: Common Nutrition Problems in Left-Behind Children (Stunting)
- Workshop 5: Common Nutrition Problems in Left-Behind Children (Iron Deficiency Anaemia)
- Workshop 6: Health Evaluation and Its Importance for Left-Behind Children

To facilitate delivery of these educational workshops for the intervention, flipcharts and folders will be developed, together with interactive lesson plans. Associated illustrations will be designed to improve caregivers’ understanding of basic nutrition and convey the importance of diversifying young LBC’s diets. The sessions will also cover the benefits of attending basic public health services. Before implementation, these materials will be field-tested for clarity and cultural appropriateness with the caregivers of LBC in similar villages not included in the sample.

The workshops, each lasting approximately 30 minutes, will be facilitated by a trained town project director and will be held bimonthly in the town hospital or health centre. The caregivers will be notified about the time, location and content of each workshop 2 weeks in advance through the village doctors and/or family planning staff. A register of attendance will be taken and recorded at each workshop to verify fulfilment of the condition for payment.

#### Setting the CCT conditionality

CCT will be used to give caregivers an incentive to attend the nutrition education workshop and bring the LBC for basic public health services. Eligible households will receive RMB 500 (equivalent to US$80) as a health grant (for bringing each left-behind child to health checkups and immunization appointments) and RMB 300 (equivalent to US$48) as a nutrition education grant (for attending six nutrition workshops). A maximum of three LBC beneficiaries will be covered by the health grant. Thus, a household with three or more LBC beneficiaries will receive RMB 1800 ([RMB500 × 3] + RMB300) (equivalent to US$288) during the intervention year if they comply with the following conditions:

1. Accompany the LBC to the town hospital and/or health centre for health checkups and growth monitoring appointments on two occasions (RMB 200 for each visit, equivalent to US$32) and for routine vaccinations (RMB 100, equivalent to US$16).
2. Attend six nutrition education workshops (RMB 50 for each session, equivalent to US$8).

#### Establishment of a local network

**Fig. 2:**
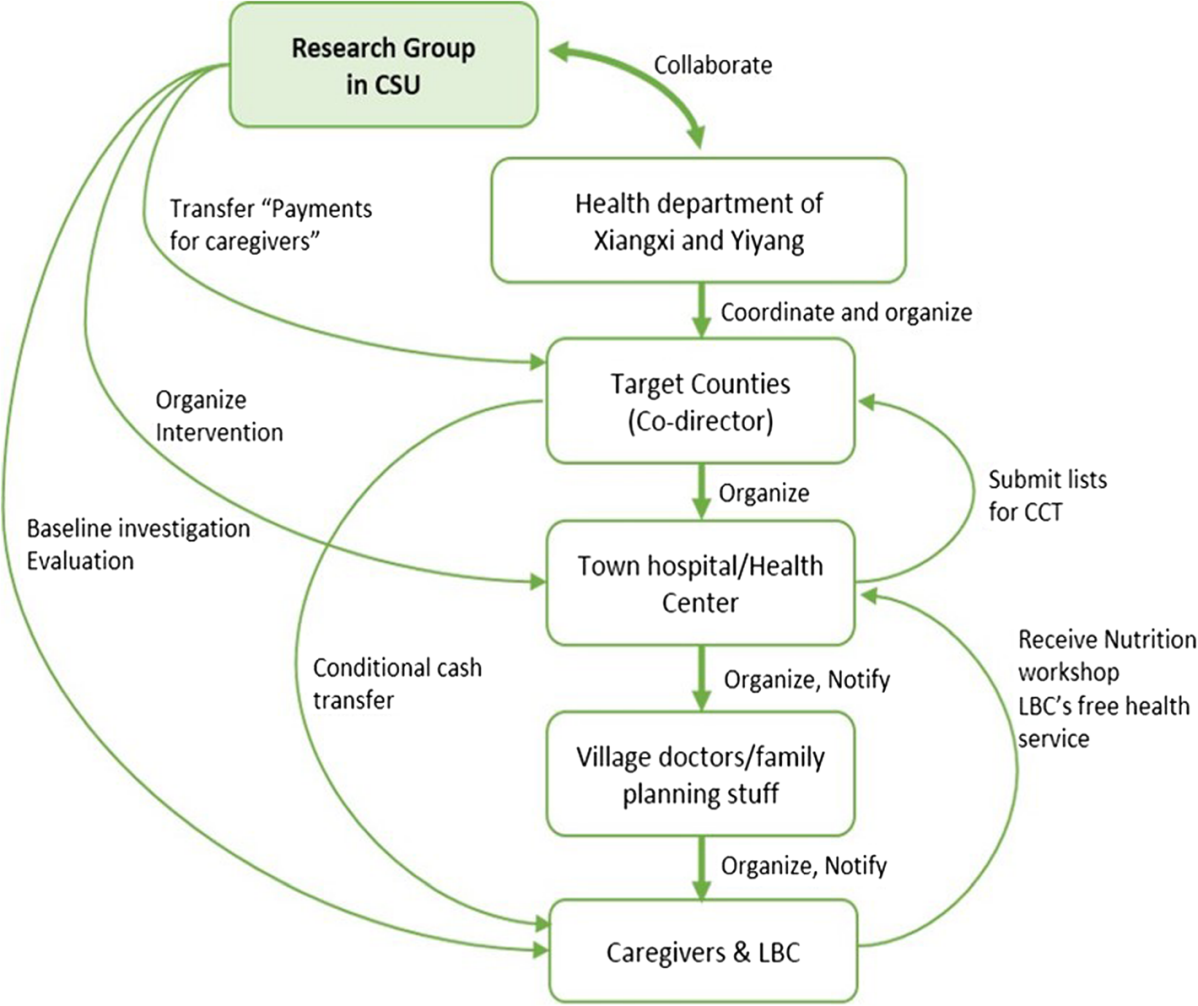
Local network for project implementation. *CCT* conditional cash transfer, *CSU* Central South University, *LBC* left-behind children

A local network will be established for project implementation (see Fig. 2). The research group in Central South University will collaborate with the health departments of Yiyang City and Xiangxi Tujia and Miao Autonomous Prefecture, and these health departments will coordinate and organise the target counties participating in the project.

#### Implementing the intervention

The intervention will be delivered at the level of the cluster (village) over a period of 1 year in villages allocated to the intervention arm. The intervention package includes CCT and nutrition education workshops. CCT will be used to give caregivers an incentive to attend the nutrition education sessions and use basic public health services that are freely available for 3–5-year-old LBC. Eligible caregivers will be invited to participate, and we may target the female caregivers in the household (grandmother or mother as a single parent of LBC). Valid identification documents and contact information of each caregiver will be submitted to the town hospital for cash transfer registration.

The cash transfer will take place bimonthly. In between payment dates, the town project director will submit to the county project coordinator a list of activities in which LBC and their caregivers participated. The project coordinator will then arrange for payment of the appropriate amount for each household. The payments will be made in cash to each caregiver in the intervention arm.

The workshop register of attendance (using valid identification for both the caregivers and the LBC) will be used to verify fulfilment of the condition for cash transfer.

### Comparator

No intervention (care as usual) will be delivered in the control villages, although the same measurements undertaken in the intervention arm will be performed at the equivalent time points for caregivers and LBC in this arm.

### Monitoring

We will form an intervention monitoring team in each county. This team will be made up of two investigators and one county health officer who will be responsible for all implementation activities in the intervention arm. They will monitor the payment systems, compliance with conditions for cash transfer, the health examination processes and the quality of the nutrition education workshops. A process monitoring visit will be undertaken every 2 months, and random spot checks will also be undertaken in between. Telephone interviews will be conducted every 2 months in a sample of four villages, including interviews with a purposive sample of caregivers, all town project directors and county project codirectors. A quarterly monitoring report will be submitted to the research team, which will review it and advise if any change is required, including provision of additional staff training if necessary.

### Outcome measurements

Measurements will be obtained at baseline and 12 months after the intervention. Children in both control and intervention villages will be invited to attend measurement sessions at the town hospital or health centre, and all caregivers will be reimbursed for their time and transportation costs for attending the measurement appointments (RMB 60 per measurement session).

### Primary outcome

The primary outcome will be the differences in the prevalence of IDA and the prevalence of stunting in the control arm compared with the intervention arm at follow-up, adjusted for baseline values. Full blood count results will be used to identify IDA, defined as mild, moderate or severe if the haemoglobin level is 110–114 g/L, 80–109 g/L or <80 g/L, respectively. Haemoglobin levels are used to diagnose anaemia, World Health Organisation (WHO) [26]. Anthropometric measurements (assessed using standardised instruments and protocols) will be used to calculate body mass index (BMI), BMI Z score, height-for-age Z score, weight-for-age Z score, weight-for-height Z score and mid-upper arm circumference. Stunting will be defined as height-for-age Z scores less than −2 SD compared with the WHO child growth standards.

### Secondary outcomes

#### Basic public health service use

We will assess whether the intervention increases access to and use of basic public health services by LBC. Particularly, we will assess the number of completed health checkups, vaccinations and growth monitoring. Reported disease prevalence and medical consultations in the previous 2 weeks will be used to compare LBC’s health needs and health care demands between the intervention and control groups.

#### Dietary change

We will assess whether the health allowance programme increases food expenditure and dietary diversity among 3–5-year-old LBC. The difference in food expenditures will be used to evaluate the impact of the intervention on food purchasing. The quantity, categories and quality of food (protein density) purchased will be calculated. The Household Dietary Diversity Score will be used to evaluate the dietary diversity between the groups. The intake of total calories, protein and some micronutrients will be estimated by use of 24-hour recall measures, which will be compared with the Chinese dietary reference intake [27]. The starchy staple ratio (SSR), which is the summary measure of household nutritional welfare, will be used to assess the impact of the change in food expenditure on household dietary composition. SSR is defined as the share of caloric availability derived from starchy staple foods (cereals and tubers). This ratio is inversely related to the importance of inexpensive starches relative to higher-quality, more expensive, micronutrient-rich foods (i.e., meat, fish, eggs and fruits).

#### Nutrition knowledge among caregivers

Evaluation of caregivers’ knowledge of LBC’s nutrition and health will be based on the average knowledge score and the average rate of correct responses. The number of attended nutrition education workshops will also be used to evaluate the impact of CCT on receiving nutrition education.

#### Cost-effectiveness

We will assess the cost-effectiveness of the programme in relation to the cost per case of IDA or stunting prevented. An incremental cost-effectiveness analysis from a health care perspective will be undertaken with a sensitivity analysis that considers the participant’s out-of-pocket costs in addition to health care costs. The primary outcome in the economic analysis will be the cost per case of IDA and the amount of stunting that is prevented. The secondary outcome will be the cost per unit increase in child public health service visits.

#### Costs

The total costs will be measured from both a health and a societal perspective. The intervention costs will include the cash transfer and the estimate of the infrastructure costs required to deliver the intervention. The costs of the staff salary, payments to participants and administrative overhead will be directly collected and calculated as a portion of the total project costs. Data on the number of staff involved and time taken to organise and manage the intervention and deliver the nutrition workshops, as well as the cost of materials to run the workshops, will be collected. The cost of the participants will also be estimated in terms of their time, travel costs (distance of travel to workshop and hospital venue and average transport costs for the distance) and any loss of income resulting from accompanying the LBC. The latter costs will be collected through interviews with participants.

#### Effectiveness

The impact of the programme will be measured in terms of changes in IDA incidence, stunting incidence and public health service use. The cost-effectiveness ratio will be calculated as follows:

1. Cost of serving one 3–5-year-old LBC with the intervention for 1 year
2. Cost of each case of stunting prevented among 3–5-year-old LBC
3. Cost for each case of IDA prevented among 3–5-year-old LBC
4. Cost for each case of use of increased public health service use among 3–5-year-old LBC

### Measurement instruments

The instruments used in this trial will include the following: information form of administrative village, LBC household questionnaires, LBC status and use of health services questionnaires, food frequency questionnaires for LBC, 24-h dietary recall for LBC, checklist to record food purchasing practices, health checkup record form and health allowance lists. The validity and reliability of all of the instruments will be assessed in the pilot study. The reliability examination will include test-retest reliability and internal consistency. The validity assessment will include structural validity, differences among LBC with regard to age and sex, differences among caregivers with various education levels, and changes after the intervention. The reliability of the anthropometric data will be checked by calculating the absolute difference between the two anthropometric measurements. If the difference is greater than 1 cm in height or 0.2 kg in weight, the measurements will be repeated.

### Sample size

The sample size calculation is based on the primary outcome to detect a difference in the IDA prevalence between the arms at follow-up. Current data suggest that the prevalence of IDA among rural children is 34.3 %; the national target is 12 % for this group. The target of the Chinese Children Development Programme 2011–2020 was IDA among rural children under 12 %. We plan to recruit 40 villages (clusters), with 20 villages each randomised to the intervention and control arms. With an average cluster size of 10 eligible children (allowing for a 33 % dropout rate) during the course of the study, at a 5 % significance level and with 80 % power, we would have sufficient power to detect a difference in the IDA prevalence of 17 % (i.e., from 34 % to 17 %), assuming a conservative estimate for an interclass correlation coefficient (ICC) of 0.1. If the ICC is as low as 0.01, this sample size will provide sufficient power to detect a difference of 13 % (i.e., from 34 % to 21 %) [28].

The pilot study will provide information on cluster sizes (i.e., the number of LBC per village). We currently assume that there are equal cluster sizes, but the variation in cluster sizes will be estimated based on the pilot study. The sample size estimate will be revised if necessary and will be used to inform our sample size calculation.

### Method of random allocation

The unit of randomisation will be the village. The local health departments of Fenghuang County and Anhua County will assist us in the random selection of villages. The baseline measurements, including anthropometric measures and blood tests, will be performed for all eligible LBC whose caregivers have consented in the 40 selected villages before randomisation. Allocation concealment will be achieved by performing all of the baseline measurements before randomisation. The random allocation sequence will be produced by an independent statistician who is not involved in the study. A balancing algorithm will be used to randomise villages to either the intervention arm or the control arm [29, 30]. Essentially, this algorithm will randomly select one of many allocation designs that will minimize the imbalance between covariates. The covariates that will be considered for inclusion in this algorithm will be the number of 3–5-year-old LBC, average annual household income, distance between village and town hospital, ethnicity, use of basic health service among 3–5-year-old LBC in the previous year, and IDA prevalence. Additionally, allocation will consider the distance between villages to avoid contamination. If two villages allocated to different arms are found to be too close to each other (<5 km), the random sequence generator will be rerun.

Blinding of the village’s allocation in this setting will not be possible. Therefore, the investigators and participants will not be blinded in this trial. However, the outcome assessment and the interim statistical analysis will be done in blinded fashion.

### Statistical methods

#### Baseline characteristics

The baseline characteristics will be summarised according to the control and intervention arms. These baseline characteristics will be summarised using their means and standard deviations, medians and interquartile ranges, or numbers and percentages as appropriate. These characteristics will include ethnicity, annual household income, number of LBC (one parent has left or both parents have left), distance between town hospital and village and the transportation cost, prevalence of IDA among 3–5-year-old LBC, and proportion of 3–5-year-old LBC using free health services (i.e., receiving free annual health examinations, vaccinations, routine blood tests, eyesight examinations, hearing screening for 3-year-olds and health evaluations) during the previous year.

#### Analyses of outcomes

The analyses of the outcomes will be performed based on the intention-to-treat principle. Therefore, all LBC will be invited back for final assessments, regardless of whether their caregivers implemented the intervention. As randomisation will be at the village (cluster) level, appropriate statistical methods to account for the clustering within villages (detailed below) will be used in the analysis. The analysis of the outcomes will include 1 year of follow-up.

The primary aim of the study is to evaluate whether the change in nutrition status differs between the arms. Stunting and IDA will be calculated based on the results of the anthropometric measurements and biochemistry tests. The secondary outcomes and use of public health services will also be compared between the two arms, including differences in vaccine coverage and attendance at health checkups, routine blood tests, and eyesight screening coverage for children aged 3 years, and hearing screening coverage. The primary analysis will be adjusted for baseline IDA or stunting prevalence only (or baseline use of public health services). Secondary analysis will adjust for both the baseline values and village-level covariates. The covariates to be included in the adjustment will be prespecified and will include household income, household size, characteristics of the caregiver (age, sex, education, career and relationship with the LBC) and LBC’s age and sex. Null hypotheses for the secondary outcomes are similar to that for the primary outcome. The analysis of the secondary outcomes will take a form similar to that described for the primary outcome.

We will estimate treatment effects using the generalized linear mixed model. The outcomes are either binary (e.g., IDA, stunting, use of public health service) or continuous (e.g., height for age, BMI), and therefore either log (Poisson model) or linear link functions will be used, with transformations as appropriate to accommodate any non-normality. To allow for clustering within villages, we will use random-effects models. We will also explore whether we need to additionally include an extra level within the random-effects models to allow for any clustering within families.

All of the model assumptions will be checked, goodness of fit will be explored and models will be selected using stepwise procedures and an analysis of deviance. The primary analysis will be a complete case analysis. However, missing data will be reported, and associations between outcomes will be explored. Depending on the nature of these associations and the extent of the missing data, sensitivity will be explored using multiple imputation. The primary outcome and primary subgroup comparisons at both time points will be considered significant at the 5 % level (95 % confidence intervals will be reported). We will report both relative risks and risk (or mean) differences as treatment effects to ensure that the magnitude of the impact is clear.

#### Planned subgroup analyses

An examination of whether any difference in outcomes between the control and intervention arms varies by the sex of the LBC, LBC age, or nutrition status at baseline will be conducted. The significance of subgroup effects will be assessed by tests of interactions of covariates and the treatment effect. The study will have low power to detect all but the largest differences.

#### Cost-effectiveness

An incremental cost-effectiveness analysis from a health care perspective will be performed, with a sensitivity analysis that consider the participant’s out-of-pocket costs in addition to health care costs. The primary outcome for the economic analysis will be cost per case of IDA and stunting that is prevented. The secondary outcome will be cost per unit increase in LBC’s public health service visits.

#### Costs

The total costs will be measured from both a health and a societal perspective. The costs of the intervention will include the cash transfer as well as an estimate of the costs of the infrastructure required to deliver the intervention.

#### Effectiveness

The impact of the programme will be measured, including changes in IDA incidence, stunting incidence and public health service use.

### Preliminary feasibility and background work

The pilot study will be conducted with four types of activities that will be involved in the final design of the main study.

1. We will conduct a systematic review of policy documents and the literature relating to health service use among rural children (LBC) since the National Basic Public Health Service Standards 2011 were issued. The findings will be used to develop the intervention strategies and deliver the evaluation indicators for the quantitative research. Structured forms and questionnaires with open- and closed-ended questions will be developed for quantitative research. Interview protocols and topic guides will also be developed. The instrument will be double-checked to identify whether it provides the information we require and whether interviewers and respondents feel comfortable with it.
2. Secondary data with characteristics of the 340 villages in Fenghuang County and 432 villages in Anhua County will be collected. A form will be completed by the village’s doctors or village family planning staff. The form will be used to gather data on the village population, number of 3–5-year-old LBC, average annual household income, distance between village and town hospital, ethnicity, use of basic public health services by 3–5-yearold LBC in the previous year, prevalence of IDA among LBC, and so forth. The sample size of the trial will be adjusted based on the prevalence of IDA.
3. The study protocol and all of the measurement instruments will be tested in two villages in Fenghuang County and two villages in Anhua County, which will be purposively selected for the pilot. The face validity and reliability of research instruments for primary data collection will be tested. The instruments will be revised if necessary before being used in the main study.
4. The feasibility of the intervention strategy will be examined. The flipcharts and other materials designed for nutrition education will be developed and then field-tested for clarity and ethnicity appropriateness with caregivers of LBC.

## Discussion

A Chinese national report indicates that the deaths of children younger than 5 years old in China which are attributable to undernutrition decreased from 22 % in 2000 to 13 % in 2010. However, this overall pattern conceals much higher rates of child undernutrition in some rural areas. As previously described, the Chinese government has implemented successful nutrition programmes targeting rural infants (younger than 3 years old). However, no suitable interventions are in place for those aged 3–5 years, a group in which undernutrition is particularly prominent.

The aim of this trial is to evaluate the provision of CCT in combination with nutrition education on improving nutritional status and development of 3–5-year-old LBC. The effect of CCT reported in the literature is not consistent across all age groups, but most CCT programmes have shown a positive impact on children’s health. The results of a recent systematic review also suggest that such interventions are promising. There are also potential benefits of cash transfer and nutrition education in our setting. The cash transfer has the potential to increase the household’s purchasing power for more and better-quality food and to reduce transportation costs incurred to access public health services for LBC. The alternative mode used in previous studies is to provide nutrition education to caregivers with the aim of increasing their awareness to modify their behaviour, thus resulting in better dietary management and increasing children’s health service use. Therefore, in this study, we propose a novel approach combining CCT with nutrition education to tackle undernutrition in LBC.

Since CCT interventions have not been assessed previously in relation to improving children’s nutritional status in China, the findings of this study could provide strong evidence for central and local government health policy decision-makers in terms of long-term implementation. The approaches could be refined based on our experience, and necessary operational adjustments could be made before further extension. Our CCT programme may also have a long-term positive impact on target participants, even after the beneficiary exits the programme. Any improvement in nutrition status will be beneficial for the LBC’s future health and development. Cash transfers can be saved and used for small investments in farming and agricultural activities (e.g., purchasing seeds for food crops, chemical fertilizer or forage), which will reduce the household economic burden for a particular time period. Furthermore, the knowledge gained from the nutrition education workshops has the potential to impact long-term behaviours.

Cashlike instruments (food stamps, vouchers and coupons) will not be used in our study, which will allow maximum flexibility for the cash to be used as needed for food or transportation to access health services for the LBC. According to our knowledge of the region, we are aware that the costs of transportation and food vary widely among the villages, which is related to the local geographic characteristics, agriculture and economic level. Currently, there is no evidence for setting a standard value for transportation coupons or food stamps for these rural areas in China. The literature on implementing CCT programmes shows that, although the use of cash (rather than vouchers or coupons) in programmes is used mainly as intended, not all of the money provided to families is spent on food. Some of the cash may be saved for future household use. The results of this study will provide evidence for whether cashlike instruments would be more appropriate for use in future nutrition interventions in China.

## Trial status

The trial started its pilot phase in Anhua and Fenghuang counties of Hunan Province in September 2014, and we 759 are currently in the process of collecting the second 760 round of data. Recruitment began in March 2015. Baseline investigation finished in May 2015. Intervention began in June 2015.

## Abbreviations

BMI: Body mass index
CCT: Conditional cash transfer
CSU: Central South University
ICC: Interclass correlation coefficient
IDA: Iron deficiency anaemia
LBC: Left-behind children
NGO: Non-governmental organisation
RMB: Chinese yuan renminbi
SSR: Starchy staple ratio
WHO: World Health Organisation
